# Management of Nasopharyngeal Carcinoma in Elderly Patients

**DOI:** 10.3389/fonc.2022.810690

**Published:** 2022-02-01

**Authors:** Wing Lok Chan, James Chung Hang Chow, Zhi-yuan Xu, Jishi Li, Wing Tung Gobby Kwong, Wai Tong Ng, Anne W. M. Lee

**Affiliations:** ^1^ Department of Clinical Oncology, The University of Hong Kong, Pokfulam, Hong Kong SAR, China; ^2^ Department of Clinical Oncology, Queen Elizabeth Hospital (QEH), Hong Kong SAR, China; ^3^ Department of Clinical Oncology, Shenzhen Hospital, University of Hong Kong, Shenzhen, China

**Keywords:** nasopharyngeal carcinoma, elderly, geriatric assessment, frailty, chemotherapy, radiotherapy

## Abstract

Nasopharyngeal cancer (NPC) is one of the most difficult cancers in the head and neck region due to the complex geometry of the tumour and the surrounding critical organs. High-dose radical radiotherapy with or without concurrent platinum-based chemotherapy is the primary treatment modality. Around 10%–15% of NPC patients have their diagnosis at age after 70. The management of NPC in elderly patients is particularly challenging as they encompass a broad range of patient phenotypes and are often prone to treatment-related toxicities. Chronologic age alone is insufficient to decide on the management plan. Comprehensive geriatric assessment with evaluation on patients’ functional status, mental condition, estimated life expectancy, comorbidities, risks and benefits of the treatment, patients’ preference, and family support is essential. In addition, little data from randomized controlled trials are available to guide treatment decisions in elderly patients with NPC. In deciding which treatment strategy would be suitable for an individual elderly patient, we reviewed the literature and reviewed the analysis of primary studies, reviews, and guidelines on management of NPC. This review also summarises the current evidence for NPC management in elderly adults from early to late stage of disease.

## Background

Nasopharyngeal carcinoma (NPC) is characterised by its unique and distinct geographical distribution, with 70% cases in east and southeast Asia. Because of its challenging location below the skull base limiting surgical accessibility, radiotherapy (RT) is the primary treatment modality. Although the classical non-keratinizing type is radiosensitive, a high-radiation dose is needed for achieving locoregional control. Furthermore, the combination of RT with concurrent plus induction or adjuvant chemotherapy is recommended for patients with advanced disease. The management of NPC in the elderly population is particularly challenging. Elderly patients are at a higher risk of toxicity from anticancer treatment due to comorbidities, as well as suboptimal nutritional status, organ function, and/or social support as compared to younger patients (1–4). There is lack of treatment guidelines for optimal management for the elderly. This review highlights the specific consideration in assessment in elderly patients with NPC and summarises the current evidence-based treatment landscape. The age distribution of NPC is unique: the relative risk increases with age and peaks at approximately 55 years of age and begins to decline at ages over 60 years. According to the Hong Kong Cancer Registry, the proportion of new cases of NPC with age ≥ 70 ranged from 10.6% to 14.4% (5).

Majority of NPC patients as a whole presented with advanced disease. Two studies showed that the stage distribution pattern of elderly NPC is similar to that of the general population (5, 6). In Hong Kong, the overall stage distribution at presentation was as follows: stage I 6.5%, stage II 14.1%, stage III 40.7%, and stage IV 29.8% (7). For patients aged ≥ 70 years, 50.6% had stage III disease while 25.2% had stage IV disease. In China, according to the National Cancer Data Base (NCDB), over 63.1% of patients aged >65 years had stage III and IV disease (8). In Singapore, similarly, 73.4% of elderly NPC patients had stage III or IV disease (9).

With advanced disease at presentation, together with presence of multiple comorbidities and suboptimal functional status, most clinicians would use less aggressive treatment in elderly patients. Previous studies reported that elderly patients with NPC had a worse treatment outcome compared with the overall population. In a matched cohort study, despite similar rates of complete locoregional response, survival of NPC patients aged ≥65 who underwent chemoradiotherapy was significantly lower than that of demographic-matched young-age control (5-year OS, 75.3% vs. 54.6%; 5-year CSS, 77.8% vs. 56.6%) (10). In the study by Huang et al. on 1,137 NPC patients aged ≥65, the 5-year OS was only 50.4%, which is lower than that of the general population of over 60% (8). Age over 75 was one of the independent prognostic factors in OS [hazard ratio (HR) 1.951, 95% confidence interval (CI) 1.552–2.453, p-value <0.001]. The study by Liang et al. also found that elderly patients had significantly worse 5-year OS compared with younger patients [5-year OS: 82.7% (age 60–<65) vs. 60.9% (age 65–<70) vs. 46.2% (age ≥70), p-value = 0.002] and progression-free survival (PFS) [5-year PFS: 74.3% (age 60–<65) vs. 60.9% (age 65–<70) vs. 46.5% (age ≥70), p-value = 0.002] (11). Sommat et al. similarly showed that age >70 had significant negative impact on OS (HR 2.40, 95% CI 1.44–4.00, p-value 0.001) and disease-free survival (DFS) (HR 1.85, 95% CI 1.02–3.36, p-value 0.043) (9).

## Challenges Faced by Elderly Patients With NPC

### Limited Data From Prospective Studies

Despite the increasing number of the elderly population with cancer, elderly individuals were often underrepresented in clinical studies. In the MAC-NPC meta-analysis, those aged ≥60 years attributed only 13% of the total cohort (12). Patients aged 70 years and above are often excluded from clinical studies which often selected participants with better performance status and general condition. Moreover, the age cut-off of “elderly” is not universally defined. Previous prospective studies on NPC selected 60–65 years as the cut-off point for the elderly NPC population. The general health in 60–65 years is improving globally, making the choice of this age cut-off arguable. Currently, the cut-off of 70 years is the most commonly adopted cut-off for defining patients as “elderly” in the field of geriatric oncology. In addition, many elderly patients do not meet the eligibility criteria of clinical trials, due to comorbidities, reduced organ functions, and/or worse performance status. The optimal treatment of elderly patients (aged ≥70) remains unclear. The existing treatment guidelines for management of NPC are based on studies on non-elderly patients, limiting the applicability in the elderly population.

### Comorbidities

Smoking is one of the risk factors for development of NPC (13). It is also associated with other comorbidities, including chronic obstructive pulmonary disease, cardiovascular disease, renal impairment, gastrointestinal disorders, and metabolic syndrome. Elderly patients with NPC, at the same time, have a higher incidence of these comorbidities. There are different validated assessment tools to evaluate comorbidities in elderly patients, e.g., Charlson Comorbidity Index (CCI) and the Adult Comorbidity Evaluation-27 (ACE-27) instrument (ACE-27). Both tools are shown in **Appendix I**. The incidence of comorbidity in elderly patients with NPC ranged from 22.4% to 58%. The study by Huang et al. reported comorbidities present in 22.4% of patients using the Charlson Comorbidity Index (CCI) (8). Ramakrishnan et al. using the Adult Comorbidity Evaluation-27 (ACE-27) instrument reported a 44% incidence of comorbidities in 59 patients with NPC, with cardiovascular and pulmonary diseases as most common (14). Guo et al. demonstrated that 44.2% patients with NPC in southern China had comorbidity using ACE-27, with the most common comorbidity being gastrointestinal disease (15). The wide difference in incidence of comorbidity is partly due to the use of different indices to measure comorbidities. ACE-27 identified more comorbidities than CCI as ACE-27 captures additional pancreatic, neuromuscular, psychiatric, and a wider range of cardiovascular diseases, alcohol and illicit drug use, and obesity information not captured by CCI (16).

Medical comorbidity has been reported to have a strong relationship with survival and outcome. Huang et al. demonstrated that presence of comorbidity was significantly associated with worse OS (8). Patients with a CCI score of 0 had significantly higher 5-year OS than patients with a CCI score of 1 or ≥2 (53.1% vs. 42.2% vs. 32.9%, p < 0.001). In the multivariate analysis, CCI was a statistically significant independent prognostic factor for the risk of death of all causes for patients with a CCI score of 1 (HR 1.242; 95% CI 1.002–1.539) or CCI score of ≥2 (HR: 1.625; 95% CI: 1.157–2.283) when compared to patients with a CCI score of 0. The study by Sze et al., which included 103 patients aged >70, revealed that ACE-27 was a significant prognostic factor for OS (5-year OS: 48.5% for ACE-27 score 0–1 vs. 20.6% for ACE score 2–3, p = 0.003) and cancer-specific survival (CSS) (5-year CCS: 71.1% for ACE-27 score 0–1 vs. 53.0% for ACE score 2–3, p = 0.02) (6). The study by Jin et al. on 126 NPC patients aged 70 or above treated with intensity-modulated radiotherapy (IMRT) showed that ACE-27 was significantly associated with survival outcomes (5-year OS: 72.9% in ACE-27 score 0–1 vs. 39.9% in ACE-27 score 2–3, p = 0.001) (17). Guo et al. also showed that comorbidity was a significant independent prognostic factor for OS and DFS. Patients with ACE-27 score > 1 had a significantly better OS compared with those with score ≤ 1 (5-year OS 81.1% vs. 59.3%, p < 0.001) and better DFS (5-year DFS: 74.1% vs. 43.9%, p < 0.001) (15). Yang et al. similarly demonstrated that a higher CCI score was associated with worse survival (18).

In addition, elderly patients usually have poor nutritional status, immobility, and cognitive decline, leading to impaired tolerability to treatment and higher risk of adverse events compared with the younger population.

### Assessment of Elderly Cancer Patient With NPC

All these considerations cautioned against using chronological age alone in risk-stratifying patients for management for NPC. Both the International Society of Geriatric Oncology (SIOG) and the American Society of Clinical Oncology (ASCO) recommend comprehensive geriatric assessment (CGA) to develop individually tailored cancer care plans for elderly patients (19, 20). CGA is regarded as the gold standard in the assessment of frailty. It is a multidimensional and multidisciplinary evaluation of elderly patients’ physical functioning (multimorbidity, mobility/falls, nutrition, polypharmacy, and sense [sight and hearing]), psychological health (cognition and mood), functional status (activities of daily living and instrumental activities of daily living), and social well-being (21). It allows the clinical team to identify risk factors such as dementia, malnutrition, and poor social support, which may compromise the tolerability of treatment, and hence individual tailoring of recommendations to optimize cancer treatment and follow-up. **Table 1** shows the important domains of assessment in elderly patients with cancer. For example, NPC patients with poor nutritional status have poor prognoses and worse overall survival (22). Early nutritional intervention can improve nutritional status, increase tolerance to cancer treatment, improve QoL, and prolong survival. Depression is another poor prognostic factor for elderly patients with NPC (23). Early management of depression would help patients to better tolerate the side effects of treatment. Patients with multiple comorbidities also have more acute toxicities in chemoradiotherapy; omission of chemotherapy may have to be considered.

**Table 1:** Important domains in geriatric assessment.

Geriatric assessment domain	Examples of evaluation tools	Suggested interventions
**Functional status**	Instrumental activities of daily living (The Katz Index of Independence in Activities of Daily Living, Lawton Instrumental Activites of Daily Living Scale), Physical performance (ECOG performance status, KPS)	Prehabilitation
**Nutrition**	Body mass index Weight loss (kilogram loss in 2 months) Swallowing problem	Dietary counselling Nutritional supplement
**Cognition**	Cognitive screening (Mini-cog), decision making capacity	Complete neuropsychological evaluation
**Comorbidities**	Number of comorbidities (e.g., Charles Comorbidity Index, ACE-27)	Referral to relevant specialties
**Polypharmacy**	Number of medications used Any inappropriate use of medications Compliance of medications Any drug interactions with anti-cancer treatment	Refer pharmacy to review the medications used
**Falls**	History of falls (number of falls in the past 6 months)	Physiotherapy, fall risk assessment, home environment modification, use of walking aids
**Life expectancy calculation**	Actuarial tables, personalized calculators	Better communication platform with patients and carers
**Treatment toxicity calculation**	Geriatric assessmet-bsed calculators, e.g., CARG toxicity tool, CARSH score	Treatment dose adjustments, need of any prophylactic treatment
**Social support**	Number of carers at home in daytime and night time Capacity of carers to take care of the patient	Referral to social worker if needed
**Psychological assessment**	Depression/anxiety (geriatric depression scale, HADS)	Referral to clinical psychologist Use of medications

ECOG, Eastern Cooperative Oncology Group; KPS, Karnofsky Performance Status; HADS, Hospital Anxiety and Depression Scale; ACE-27, Adult Comorbidity Evaluation-27; CARG, The Cancer and Aging Research Group; CRASH, Chemotherapy Risk Assessment Scale for High-Age Patients.

Although CGA is recommended in different international oncology guidelines, it is not widely implemented because it is time consuming and there is a shortage of trained staff. Geriatric screening tools have been developed and can be used to identify those who will benefit most from a geriatric assessment. To date, Geriatric-8 (G8) and Vulnerable Elders Survey-13 (VES-13) were the two most commonly used tools for identifying patients who need CGA (24). The details of G8 and VES-13 are shown in **Appendix 3** and **4**. Studies also showed that G8 could predict survival. The study by Pottel et al. (25) demonstrated that vulnerability as classified by G8 was an independent predictor for survival after curative chemoradiotherapy for elderly patients with head and neck cancer (25). Ishii et al. also showed that an abnormal G8 score in elderly patients with head and neck cancer was significantly associated with a shorter survival, higher 30-day mortality, and all-complication rates (26). These studies showed that the geriatric screening tool not only can help identify those patients who need comprehensive assessment but also can predict complication and treatment outcome.

## Treatment of NPC in Elderly Patients

### Definitive Treatment in Non-Metastatic Disease

The current standard of care for non-metastatic NPC is high-dose radiotherapy using the intensity-modulated technique (IMRT) and incorporation of systemic chemotherapy in patients with locoregionally advanced tumours. Despite the improvement of radiotherapy techniques, treatment of NPC remains a harsh journey even for young and fit individuals.

**Figure 1:**
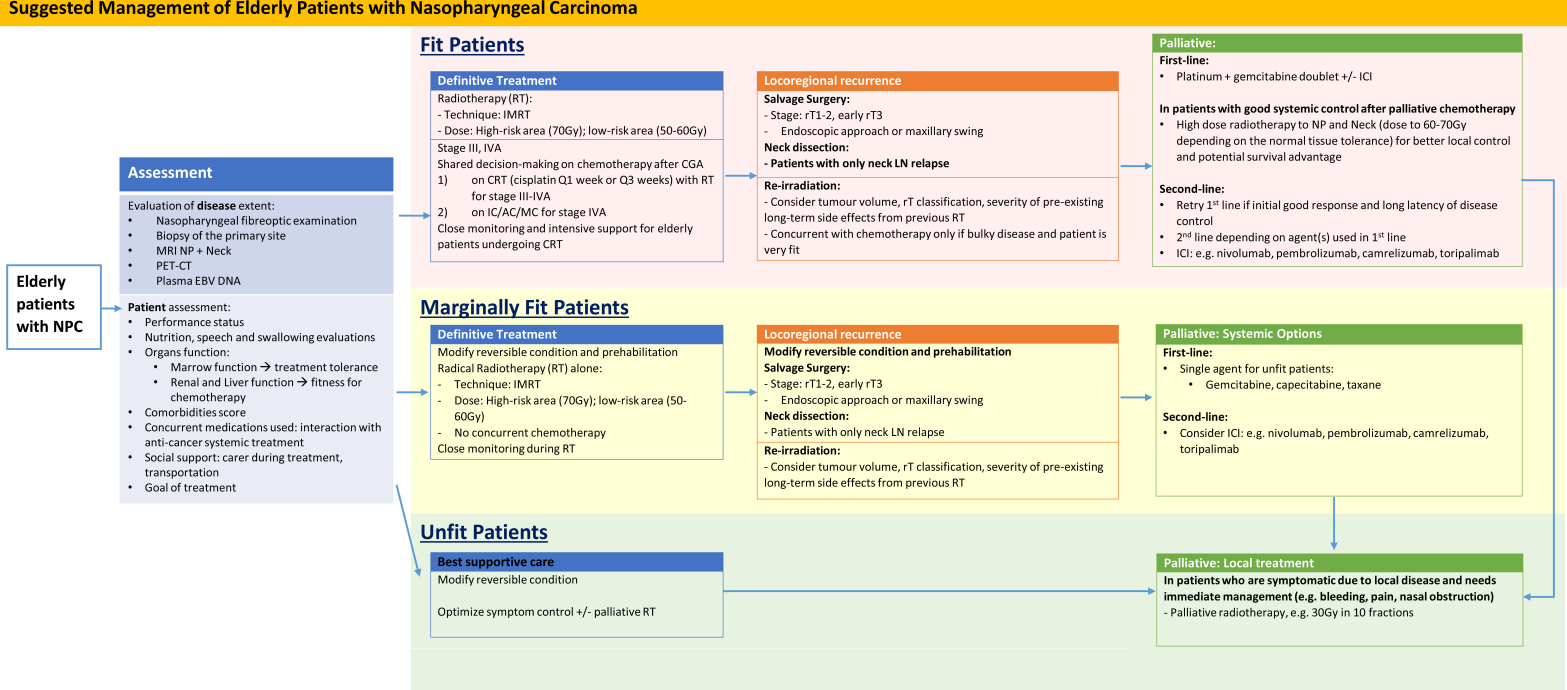
Suggested Management of Elderly Patients with Nasopharyngeal Carcinoma. AC, adjuvant chemotherapy; CRT, concurrent chemoradiation; IC, induction chemotherapy; ICI, immune checkpoint inhibitors; IMRT, intensity modulated radiotherapy; LN, lymph node; MC, metronomic chemotherapy; NP, Nasopharynx; RT, Radiotherapy.

We have performed a literature review on the management plan of elderly patients with NPC. A suggested management flow is also shown in **Figure 1**.

#### Radiotherapy

NPC is highly sensitive to ionising radiation; treatment outcomes have been excellent in contemporary series in the era of IMRT (27). Past studies on RT with the 3D conformal technique showed that elderly NPC patients had poor tolerance to high-dose radiotherapy. Sze et al. showed that patients aged ≥70 had significantly higher rates of grade 3–4 acute radiation mucositis (68% vs. 57.6%) and dermatitis (22.3% vs. 12.7%) than the younger counterpart (6). Furthermore, elderly patients had a significantly higher 90-day mortality rate compared with younger patients (7.8% vs. 1.2%, p < 0.001). This early mortality was associated with poor comorbidities: patients with a pretreatment Adult Comorbidity Evaluation-27 (ACE-27) score of 2–3 have a 15-fold higher risk of early deaths than those scored <2.

With advances in the radiotherapy technique, intensity-modulated radiotherapy (IMRT) or tomotherapy has become the standard RT technique for NPC. IMRT provides excellent tumour target coverage and allows the delivery of a high dose to the target with significant sparing of the organs at risk, e.g., salivary glands, brainstem, and cranial nerves (28, 29). Studies demonstrated that IMRT achieved better local control with fewer late toxicities compared with the 3D conformal technique. Recent studies demonstrated that elderly NPC patients treated with IMRT had similar toxicity profiles compared with others. Only few patients could not complete radiotherapy, with incidence ranging from 0% to 2.7% (9, 30). The most common severe toxicities (grades 3–4) were dermatitis and mucositis (9, 11).

With improving survival following contemporary definitive treatments, late complications have become an important and relevant domain in survivorship care. A retrospective matched cohort study did not identify a significant difference in the incidences of late radiotherapy complications between elderly and young NPC patients. Specifically, advanced age did not increase the risk of radiation-associated sensorineural hearing loss (31, 32), neurocognitive decline (33), cranial neuropathy (34, 35), and impairment in overall quality of life (32, 36, 37). Interestingly, elderly NPC patients even seemed to be less susceptible to develop post-radiation hypothyroidism (38, 39). On the contrary, elderly NPC patients are more prone to develop post-radiation xerostomia and second malignancy (40, 41). In a recent territory-wide study in Hong Kong, the risk of second cancer in post-IMRT NPC patients aged >60 was 40% higher compared with the demographic-matched general population. The absolute excess cancer risk in this elderly age group was up to 60 per 100,000 person-years at risk. As such, continual surveillance for radiation-associated second cancer is warranted in elderly NPC survivors (34).

Despite serious concern about the higher rates of early mortality and acute radiation toxicities, currently there is a lack of clinical evidence to support de-escalation of radiation dose or volume for elderly NPC patients. Radiotherapy planning for elderly NPC should follow international guidelines on target delineation and dose prioritization, maintaining prescribed radiation doses to high-risk and low-risk at 70 and 50–60 Gy, respectively (42, 43). A retrospective study using simultaneous integrated boost to gross nasopharyngeal tumour to 68 Gy in 30 fractions showed a satisfactory long-term outcome in elderly NPC patients, but 20% of patients had unplanned radiotherapy interruptions due to high-grade mucositis and hematologic toxicities (44). Compared with IMRT, intensity-modulated proton has demonstrated dosimetric advantages and a decreased rate of gastrostomy tube insertion with therapy in localised NPC (45, 46). However, its long-term therapeutic value and cost effectiveness in elderly patients remain to be elucidated by prospective comparative research.

#### Chemotherapy

Since the first report of the milestone Intergroup 0099 showing survival benefit by concurrent-adjuvant chemotherapy in the late 1990s, incorporating systemic chemotherapy has become the standard recommendation for locoregionally advanced NPC. The MAC-NPC meta-analysis which combined results of 19 trials has concluded a 6% absolute improvement in 5-year OS with the addition of chemotherapy to definitive radiotherapy (12). Both the latest ESMO-EURACAN and CSCO-ASCO guidelines recommend concurrent plus induction or adjuvant chemotherapy for advanced-stage diseases (47, 48).

When applying clinical evidence to elderly NPC patients, it must be emphasized that this population is highly under-represented in most clinical trials. According to population incidence data from the Hong Kong Cancer Registry, 44.7% of NPC patients diagnosed between 2009 and 2018 were aged ≥60 years (7), whereas the same age group only constituted 13.0% of the 4,806 patients included in MAC-NPC (12).

The therapeutic benefit of concomitant chemotherapy in elderly NPC patients has remained uncertain. Retrospective data on elderly patients (age ≥60 or ≥65) with advanced NPC suggested improvements in survival outcomes with the addition of chemotherapy to 2-dimensional radiotherapy, particularly in patients with low pretreatment comorbidities (10, 49). Nevertheless, in the MAC-NPC meta-analysis, a clear decrement in the hazard ratios for chemotherapy on OS was observable as age increases: 0.72 for age <50, 0.79 for age 50–59, and 0.89 for age ≥60, suggesting a diminished therapeutic advantage for chemotherapy in elderly NPC patients (12). This observation is in line with that of the MACH-HC meta-analysis in head and neck squamous cell carcinoma, in which no clear benefit of chemo-radiotherapy was found in patients aged ≥70, and the authors have recommended concomitant chemotherapy to be weighed carefully in the elderly age group (50). In the contemporary era of IMRT, multiple retrospective studies which focused in elderly NPC patients reported higher toxicities with concomitant chemotherapy, e.g., myelosuppression, nausea, vomiting, and mucositis (5, 9, 17, 44, 51). Hence, consideration of adding chemotherapy for elderly patients should be individualized.

Cisplatin has remained the core cytotoxic agent for treating NPC. Despite its well-proven efficacy, cisplatin is notorious for causing ototoxicity, nephrotoxicity, and peripheral neuropathy, all of which are especially worrisome for elderly patients with limited organ reserve. Expert consensus on head and neck cancer patients aged >70 at high risk of cisplatin toxicities raised the suggestion that extra care should be taken before administration (52). Understanding that a dichotomized cut-off by chronological age is oversimplified, CGA should be considered before using cisplatin. In selected elderly NPC patients with good organ functions, concomitant chemotherapy may still be a reasonable option after careful comorbidity assessment. In one study, the benefit of concomitant chemotherapy was confined to patients with ACE-27 scores of <2, whereas all patients with ACE-27 score ≥2 who underwent chemoradiotherapy experienced grade 3–4 acute toxicities (10).

Concurrent cisplatin is most commonly administered in a schedule of 100 mg/m^2^ every 3 weeks. Randomized studies have demonstrated similar efficacy of the weekly 30–40-mg/m^2^ regimens, which require less fluid pre-hydration and potentially less renal and otologic toxicities (53, 54). However, preliminary data from a phase III non-inferiority trial in NPC demonstrated significantly higher rates of grade 3–4 thrombocytopenia and leukopenia with the weekly regimen than the tri-weekly regimen (55). Although elderly patients could attain a higher cumulative cisplatin dose with the weekly regimen than the tri-weekly regimen (median, 240 vs. 160 mg/m^2^) (10), prospective evidence on the safety profile of the weekly cisplatin regimen in the elderly population is lacking.

Several studies have compared alternative platinum derivatives with conventional cisplatin in advanced NPC. Carboplatin, nedaplatin, and lobaplatin have been independently compared with cisplatin in phase III clinical trials, in which all have demonstrated non-inferior progression-free survival, with improved quality of life and less ototoxicity, nephrotoxicity, and emetogenicity (56–58). However, the upper-bound age limits for patients in these trials were set at 70, 65, and 60 years, respectively. It has therefore remained uncertain whether the efficacy and favourable toxicity profiles of these agents would be maintained in the elderly NPC population. Nimotuzumab, an anti-epidermal growth factor antibody, has also been studied in elderly NPC in one retrospective study (59). Nimotuzumab demonstrated encouraging efficacies and tolerability but incurred relatively high rates of grade 3–4 leukopenia and mucositis, when used with or without concomitant cisplatin in advanced elderly NPC patients (≥60 years old) undergoing definitive radiotherapy. Prospective phase II trials are ongoing to investigate this approach, with special focus in the elderly NPC population (ClinicalTrials.gov Identifier: NCT03025958, NCT03915132).

Based on the landmark Intergroup 0099 study and three subsequent confirmatory trials, definitive chemoradiotherapy followed by 3 cycles of 5-fluorouracil and cisplatin in the adjuvant phase is one of the standard recommendations for advanced NPC (60–63). However, as patients are still experiencing significant acute toxicities after chemoradiotherapy, the adjuvant component of this regimen is often poorly tolerated, with only approximately 60% of patients being able to complete all three cycles. Administration of adjuvant chemotherapy is particularly challenging in elderly patients (9, 17, 51). A recent study showed that metronomic capecitabine (650 mg/m^2^ body surface area twice a day) for 1 year, compared with observation, significantly improved failure-free survival in patients with high-risk locoregionally advanced NPC (3-year failure-free survival: 85.3% vs. 75.7%, HR 0.50, 95% CI 0.32–0.79, p = 0.0023) with a manageable safety profile (64). However, the study did not include patients aged >65 years.

In recent years, several phase III clinical trials have demonstrated survival advantages of induction chemotherapy before definitive chemoradiotherapy in NPC (65–67). However, elderly patients were excluded in these trials due to anticipated concern about tolerance. In a propensity score-matched analysis focused on NPC patients aged ≥60, the addition of induction chemotherapy has no significant impact on survival compared to chemoradiotherapy alone but led to worse hematologic toxicities and stomatitis (68). With the current lack of evidence, the therapeutic benefit of concurrent with/without adjuvant or induction chemotherapy in elderly NPC patients remains uncertain, the risks should be duly discussed, and close monitoring and intensive support should be provided for elderly patients treated with additional chemotherapy.

### Locoregional Recurrent NPC

Approximately 10%–20% of NPC patients will develop locoregional recurrence after definitive radiotherapy. Both surgical resection and re-irradiation are potential treatment options; the former is the modality of choice if expertise is available and a clear surgical margin is achievable (69). However, the toxicity and complication rates with either approach are high, especially in elderly patients who have suboptimal pre-morbid conditions.

Salvage surgery for locally recurrent NPC traditionally adopts open approaches, which result in considerable functional disability and cosmetic morbidities (70, 71). For early local recurrences, endoscopic nasopharyngectomy is increasingly being adopted as a less invasive but equally effective salvage option (72, 73). In a recent multicentre randomised phase 3 trial which enrolled only patients aged <70, endoscopic nasopharyngectomy was shown to improve survival in selected rT1–2 or early rT3 recurrent NPC patients compared with re-irradiation (3-year OS, 85.8% vs. 68.0%, p = 0.0015) (74). Of note, half of the mortality in the re-irradiation arm was due to treatment complications, where it was possibly associated with the unconventional adoption of elective clinical target volumes and hypofractionated radiotherapy regimes. For operable tumours in physically fit patients, chronological age on its own should not be the sole factor to preclude salvage surgery for recurrent NPC, as case series have supported its feasibility in elderly patients beyond the age of 70 (73, 75).

For patients in whom salvage surgery is not feasible, re-irradiation to the nasopharynx is a standard of care; a 5-year local control rate of 80%–85% has been reported for recurrent stage I–II diseases (76, 77). However, the cumulative radiation injury to normal tissues is associated with high rates of morbidity and mortality even in young and fit patients. In one meta-analysis, a pooled grade-5 toxicity rate of up to 33% has been reported (78); lethal pharyngeal mucositis, nasal haemorrhage, or radiation encephalopathy was observed despite stringent eligibility criteria in a clinical trial setting (74). Old age is an adverse prognostic factor for survival and an independent risk factor for lethal nasopharyngeal necrosis after NPC re-irradiation (75, 79–81). Extra caution on patient selection is required before starting elderly patients with radical re-irradiation. Apart from pretreatment CGA as in primary radiotherapy, other factors to consider include the time interval from primary radiotherapy, tumour volume, rT classification, and the severity of preexisting late toxicities (80). There is yet no concrete evidence on the benefit of integrating systemic therapy to re-irradiation, but extrapolating from the studies on primary treatment, majority of centres would recommend combined modality for locoregionally advanced recurrent NPC (69). Patient tolerance to chemotherapy is typically poor in the re-irradiation setting, with a high rate of discontinuation and treatment-related toxicities even in young individuals (82). The exact risk–benefit ratio is even more uncertain for elderly patients; routine addition of chemotherapy is generally not recommended.

### Metastatic NPC

In the current era of IMRT which offers excellent locoregional control, distant metastasis has become the major mode of relapse for localised NPC (3). In addition, approximately 10% of NPC patients present with *de novo* distant metastases (83). For platinum-sensitive metastatic disease, first-line palliative chemotherapy of choice is gemcitabine and cisplatin, which has a high response rate of 64% and confers a median progression-free survival of 7 months (84). However, this regimen is associated with a 43% rate of grade 3–4 toxicities, which were predominantly hematologic. Given the limited marrow and renal reserve in elderly patients, delivery of this cisplatin-based doublet represents a clinical challenge. Recent randomized evidence has demonstrated further improvement in PFS with the addition of camrelizumab to gemcitabine and cisplatin; however, whether elderly patients could tolerate this intensive treatment remains questionable (85). Alternatively, monotherapy such as gemcitabine, capecitabine, and docetaxel had reported response rates ranging between 24% and 48% (86–89). The choices among these active agents should be individualized by weighing the differences in side effect profiles, patient comorbidities, renal versus hepatic reserves, and the route and frequency of administration. For patients with favourable response to platinum-doublet chemotherapy, a randomized trial has demonstrated additional survival advantage with sequential high-dose locoregional radiotherapy (90). Whether elderly patients can derive the same benefit remains uncertain, especially if dose reduction, substitution by carboplatin, and/or de-escalation to monotherapy has to be adopted. Given their high vulnerability to acute radiation toxicities, sequential locoregional therapy should only be considered in highly selective elderly patients who have exceptional fitness and have achieved excellent tumour response to induction chemotherapy.

Recently, immune checkpoint inhibition has also emerged as another key pillar in palliative management of metastatic NPC. Multiple agents including pembrolizumab, nivolumab, camrelizumab, and toripalimab have demonstrated encouraging activities (91–95). Safety profiles of these agents were favourable, with grade 3–4 immune-related toxicities of approximately 10%. Elderly patients, however, were again underrepresented in all these trials. While tolerance to immune checkpoint inhibition in elderly patients was shown to be similar to younger individuals, efficacy may be lower in elderly patients due to the deteriorating immune system (96). Current clinical evidence on immune checkpoint inhibitors in NPC is emerging; subgroup analyses in comparative phase III trials may help to clarify this issue in NPC.

## Future Directions—Integrating Geriatric Assessment/Interventions Into the Management of NPC in Elderlies

Despite the new advances in the management of NPC, elderly patients were often underrepresented in clinical trials. Elderly patients are of a heterogeneous group, ranging from fit to frail, and individualised decisions are always needed. There are emerging data showing that a combination of geriatric interventions can improve clinical outcomes. The randomised controlled trial by Li et al. randomised 613 patients aged 65 years or older planned for new chemotherapy into multidisciplinary geriatric assessment–driven intervention (GAIN) and standard-of-care (SOC) arms (97). Patients in the GAIN arm had a lower incidence of grade 3 or higher chemotherapy-related toxic effects compared with the SOC arm (50.5% vs. 60.6%, p = 0.02). There was also a significant increase in the completion of advance directives in the GAIN arm (28.4% vs. 13.3%, p < 0.001). Another study by Mohile et al. which included 718 patients aged 70 years or older with advanced cancer demonstrated that integration of geriatric interventions into usual care reduced the incidence of grade 3–5 toxicities (51% vs. 71%, p = 0.0001) and incidence of falls (12% vs. 21%, p = 0.0035) (98). These studies demonstrated that the integration of geriatric interventions in cancer management in elderly patients certainly improved the clinical outcome and reduced toxicities.

A multicentre, randomised controlled trial on the impact of CGA on survival, function, and nutritional status in elderly patients with head and neck cancer (EGeSOR; Trial registration: NCT02025062) is now ongoing in 13 sites in France (99). The interventions in the study include 1) the CGA conducted by a geriatrician before cancer treatment, 2) participation of the same geriatrician in cancer treatment selection, 3) a standardised geriatric therapeutic intervention designed by the same geriatrician, and 4) geriatric follow-up for 24 months. The results of this study to demonstrate the direct clinical benefit of CGA on outcomes of older patients with head and neck cancer are eagerly awaited.

Besides optimizing the reversible condition with geriatric interventions, individualised treatment plans for fit and unfit elderly patients need to be considered. There are studies investigating if escalating or de-escalating treatment regimen is suitable for older patients. For example, the ongoing phase 3 GORTEC ELAN-RT trial (NCT01864850) compares the efficacy and safety of standard radiotherapy (70 Gy in 35 fractions over 7 weeks) with hypofractionated split course radiotherapy (30 Gy/10 fractions, with a 2-week gap for toxicity management, followed by 25 Gy/10 fractions) in vulnerable elderly patients with head and neck squamous cell carcinoma planned for radical radiotherapy (100). Another prospective study, the ELAN (Elderly head and Neck cancer) FIT and UNFIT, which was a prospective study, first classified patients aged 70 or above with recurrent or metastatic head and neck squamous cell cancer as fit and unfit (101). Unfit patients were randomised to weekly methotrexate or biweekly cetuximab. The study showed that patients with performance status ECOG 2 did not benefit from systemic treatment, and there was no efficacy difference in methotrexate and cetuximab. Similar studies on individualised management plans for elderly patients with NPC, who form a heterogeneous group from fit to frail, are warranted.

## Conclusion

In conclusion, elderly patients form a heterogeneous group in terms of their health condition, performance status, physical reserve, and social support. Unfortunately, there is a paucity of data and lack of guidelines on the management of NPC in elderly people. Moreover, most of the existing data are non-randomized. In all elderly NPC patients, pretreatment evaluation with geriatric assessment is essential to evaluate their comorbidities, physical functioning, psychological well-being, and social support. Efforts should be taken to identify risk factors for treatment-related toxicities, and modifiable factors should be controlled before treatment. Currently, similar to other adult NPC patients, IMRT is the standard of treatment in elderly NPC patients. The decision on adding chemotherapy concurrently with RT should be considered carefully because of the limited efficacy data and potential toxicities from chemotherapy. In a metastatic setting, the choice of anticancer treatment should be individualized according to the patients’ preference, general condition, and comorbidities. A multidisciplinary approach, early palliative care and good communication on treatment goals are essential to support the elderly patients and their families through the treatment pathway.

## Author Contributions

WC, JC, and WN contributed to the article writing. WK, AL, JL, and ZX edited the article. AL contributed the idea of this article writing. All authors contributed to the article and approved the submitted version.

## Conflict of Interest

The authors declare that the research was conducted in the absence of any commercial or financial relationships that could be construed as a potential conflict of interest.

## Publisher’s Note

All claims expressed in this article are solely those of the authors and do not necessarily represent those of their affiliated organizations, or those of the publisher, the editors and the reviewers. Any product that may be evaluated in this article, or claim that may be made by its manufacturer, is not guaranteed or endorsed by the publisher.
