# Supplementary material for: Management of Nasopharyngeal Carcinoma in Elderly Patients
Source: Front Oncol. 2022 Feb 1;12:810690. doi: 10.3389/fonc.2022.810690 (PMC8844547; doi:10.3389/fonc.2022.810690)
Supplement: Supplementary file 1 [file DataSheet_1.docx]

**Appendix 1: Charlson Comorbidity Index Scoring System**

| **Charlson Comorbidity Index Scoring System** | |
| --- | --- |
| Score | Condition |
| 1 | Myocardial infraction (history, not ECG changes only) |
|  | Congestive heart failure |
|  | Peripheral vascular disease (includes aortic aneurysm ≥6cm) |
|  | Cerebrovascular disease: CVA with mild or no residua or TIA |
|  | Dementia |
|  | Chronic pulmonary disease |
|  | Connective tissue disease |
|  | Peptic ulcer disease |
|  | Mild liver disease (without portal hypertension, includes chronic hepatitis) |
|  | Diabetes without end-organ damage (excludes diet-controlled alone) |
| 2 | Hemiplegia |
|  | Moderate or severe renal disease |
|  | Diabetes with end-organ damage (retinopathy, neuropathy, nephropathy, or brittle diabetes) |
|  | Tumour with metastases (exclude if >5y from diagnosis) |
|  | Leukaemia (acute or chronic) |
|  | Lymphoma |
| 3 | Moderate or severe liver disease |
| 6 | Metastatic solid tumour |
|  | AIDS (not just HIV positive) |
| NOTE. For each decade > 40 years of age, a score of 1 is added to the above score.  Abbreviations: ECG, electrocardiogram; CVA, cerebrovascular accident; TIA, transient ischemic attack; AIDS, acquired immunodeficiency syndrome; HIV, human immunodeficiency virus | |

**Appendix 2: Adult Comorbidity Evaluation-27**

**Adult Comorbidity Evaluation-27**

Identify the important medical comorbidities and grade severity using the index.

Overall Comorbidity Score is defined according to the highest ranked single ailment, except in the case where two or more Grade 2 ailments occur in different organ systems.

In this situation, the overall comorbidity score should be designated Grade 3.

| Cogent comorbid ailment | Grade 3  Severe Decompensation | Grade 2  Moderate Decompensation | Grade 3  Mild Decompensation |
| --- | --- | --- | --- |
| **Cardiovascular System** | | | |
| Myocardial Infarct | 🞏 MI ≥ 6 mos. | 🞏 MI > 6 mos. ago | 🞏 MI by ECG only, age undetermined |
| Angina / Coronary Artery Disease | 🞏 Unstable angina | 🞏 Chronic exertional angina  🞏 Recent (≤ 6 mos.) Coronary Artery Bypass Graft (CABG) or Percutaneous Transluminal Coronary Angioplasty (PTCA)  🞏 Recent (≤ 6 mos.) coronary stent | 🞏 ECG or stress test evidence or catherization evidence of coronary disease without symptoms  🞏 Angina pectoris not requiring hospitalization  🞏 CABG or PTCA (>6 mos.)  🞏 Coronary stent (>6 mos.) |
| Congestive Heart Failure (CHF) | 🞏 Hospitalized for CHF within past 6 months  🞏 Ejection fraction <20% | 🞏 Hospitalized for CHF >6 mos. Prior  🞏 CHF with dyspnea which limits activities | 🞏 CHF with dyspnea which has responded to treatment  🞏 Exertional dyspnea  🞏 Paroxysmal Nocturnal Dyspnea (PND) |
| Arrhythmias | 🞏 Ventricular arrhythmia ≤ 6 mos. | 🞏 Ventricular arrhythmia > 6 mos.  🞏 Chronic atrial fibrillation or flutter  🞏 Pacemaker | 🞏 Sick Sinus Syndrome  🞏 Supraventricular tachycardia |
| Hypertension | 🞏 DBP ≥ 130mm Hg  🞏 Severe malignant papilledema or other eye changes  🞏 Encephalopathy | 🞏 DBP 115-129 mm Hg  🞏 DBP 90-114 mm Hg while taking antihypertensive medications  🞏 Secondary cardiovascular symptoms: vertigo, epistaxis, headaches | 🞏 DBP 90-114 mm Hg while not taking antihypertensive medications  🞏 DBP <90mm Hg while taking antihypertensive medications  🞏 Hypertension, not otherwise specified |
| Venous Disease | 🞏 Recent PE (≤ 6 mos.)  🞏 Use of venous filter for PE’s | 🞏 DVT controlled with Coumadin or heparin  🞏 Old PE > 6 mos. | 🞏 Old DVT no longer treated with Coumadin or Heparin |
| Peripheral Arterial Disease | 🞏 Bypass or amputation for gangrene or arterial insufficiency < 6 mos. ago  🞏 Untreated thoracic or abdominal aneurysm (≥6 cm) | 🞏 Bypass or amputation for gangrene or arterial insufficiency > 6 mos. ago  🞏 Chronic insufficiency | 🞏 Intermittent claudication  🞏 Untreated thoracic or abdominal aneurysm (< 6cm)  🞏 s/p abdominal or thoracic aortic aneurysm repair |
| **Respiratory System** | | | |
|  | 🞏 Marked pulmonary insufficiency  🞏 Restrictive Lung Disease or COPD with dyspnoea at rest despite treatment  🞏 Chronic supplemental O2  🞏 CO2 retention (pCO2 > 50 torr)  🞏 Baseline pCO2 < 50 torr  🞏 FEVI (<50%) | 🞏 Restrictive Lung Disease or COPD (chronic bronchitis, emphysema, or asthma) with dyspnoea which limits activities  🞏 FEVI (51%-65%) | 🞏 Restrictive Lung Disease or COPD (chronic bronchitis, emphysema, or asthma) with dyspnoea which has responded to treatment  🞏 FEVI (66%-80%) |
| **Gastrointestinal System** | | | |
| Hepatic | 🞏 Portal hypertension and/or oesophageal bleeding ≤ 6 mos. (Encephalopathy, Ascites, Jaundice with total Bilirubin >2) | 🞏 Chronic hepatitis, cirrhosis, portal hypertension with moderate symptoms “compensated hepatic failure” | 🞏 Chronic hepatitis or cirrhosis without portal hypertension  🞏 Acute hepatitis without cirrhosis  🞏 Chronic liver disease manifested on biopsy or persistently elevated bilirubin (>3mg/dl) |
| Stomach/Intestine | 🞏 Recent ulcers (≤ 6 mos.) requiring blood transfusion | 🞏 Ulcers requiring surgery or transfusion > 6 months ago | 🞏 Diagnosis of ulcers treated with meds  🞏 Chronic malabsorption syndrome  🞏 Inflammatory bowel disease (IBD) on meds or h/o with complication and/or surgery |
| Pancreas | 🞏 Acute or chronic pancreatitis with major complications (phlegmon, abscess, or pseudocyst) | 🞏 Uncomplicated acute pancreatitis  🞏 Chronic pancreatitis with minor complications (malabsorption, impaired glucose tolerance, or GI bleeding) | 🞏 Chronic pancreatitis w/o  complications |
| **Renal System** | | | |
| End-stage renal disease | 🞏 Creatinine > 3 mg% with multi-organ failure, shock, or sepsis  🞏 Acute dialysis | 🞏 Chronic Renal Insufficiency with creatinine >3 mg%  🞏 Chronic dialysis | 🞏 Chronic Renal Insufficiency with creatinine 2-3 mg%. |
| **Endocrine System (Code the comorbid ailments with the (*) in both the Endocrine system and other organ systems if applicable)** | | | |
| Diabetes Mellitus | 🞏 Hospitalization ≤ 6 months for DKA  🞏 Diabetes causing end-organ failure  🞏 retinopathy  🞏 neuropathy  🞏 nephropathy*  🞏 coronary disease*  🞏 peripheral arterial disease* | 🞏 IDDM without complications  🞏 Poorly controlled AODM with oral agents | 🞏 AODM controlled by oral agents only |
| **Neurological System** | | | |
| Stroke | 🞏 Acute stroke with significant neurological deficit | 🞏 Old stroke with neurologic residual | 🞏 Stroke with no residual  🞏 Past or recent TIA |
| Dementia | 🞏 Severe dementia requiring full support for activities of daily living | 🞏 Moderate dementia (not completely self-sufficient, needs supervising) | 🞏 Mild dementia (can take care of self) |
| Paralysis | 🞏 Paraplegia or hemiplegia requiring full support for activities of daily living | 🞏 Paraplegia or hemiplegia requiring wheelchair, able to do some self-care | 🞏 Paraplegia or hemiplegia, ambulatory and providing most of self-care |
| Neuromuscular | 🞏 MS, Parkinson’s, Myasthenia Gravis, or other chronic neuromuscular disorder and requiring full support for activities of daily living | 🞏 MS, Parkinson’s, Myasthenia Gravis, or other chronic neuromuscular disorder, but able to do some self-care | 🞏 MS, Parkinson’s, Myasthenia Gravis, or other chronic neuromuscular disorder, but ambulator and providing most of self-care |
| **Psychiatric** | | | |
|  | 🞏 Recent suicidal attempt  🞏 Active schizophrenia | 🞏 Depression or bipolar disorder uncontrolled  🞏 Schizophrenia controlled w/ meds | 🞏 Depression or bipolar disorder controlled w/ medication |
| Rheumatologic (Incl. Rheumatoid Arthritis, Systemic Lupus, Mixed Connective Tissue disorder, Polymyositis, Rheumatic Polymyositis) | | | |
|  | 🞏 Connective Tissue Disorder with secondary end-organ failure (renal, cardiac, CNS) | 🞏 Connective Tissue Disorder on steroids or immunosuppressant medications | 🞏 Connective Tissue Disorder on NSAIDS or no treatment |
| **Immunological System (AIDS should not be considered a comorbidity for Kaposi's Sarcoma or Non-Hodgkin's Lymphoma)** | | | |
| AIDS | 🞏 Fulminant AIDS w/KS, MAI, PCP (AIDS defining illness) | 🞏 HIV+ with h/o defining illness. CD4+ < 200/µL | 🞏 Asymptomatic HIV+ patient.  🞏 HIV+ w/o h/o AIDS defining illness. CD4+ > 200/µL |
| **Malignancy (Excluding Cutaneous Basal Cell Ca., Cutaneous SCCA, Carcinoma in-situ, and Intraepithelial Neoplasm)** | | | |
| Solid Tumour including melanoma | 🞏 Uncontrolled cancer  🞏 Newly diagnosed but not yet treated  🞏 Metastatic solid tumour | 🞏 Any controlled solid tumour without documented metastases, but initially diagnosed and treated  within the last 5 years | 🞏 Any controlled solid tumour without documented metastases, but initially diagnosed and treated >5 yr. ago |
| Leukaemia and Myeloma | 🞏 Relapse  🞏 Disease out of control | 🞏 1st remission or new dx <1yr  🞏 Chronic suppressive therapy | 🞏 H/o leukaemia or myeloma with last Rx > 1 yr. prior |
| Lymphoma | 🞏 Relapse | 🞏 1st remission or new dx <1yr  🞏 Chronic suppressive therapy | 🞏 H/o lymphoma w/ last Rx >1 yr. prior |
| **Substance Abuse (Must be accompanied by social, behavioural, or medical complications)** | | | |
| Alcohol | 🞏 Delirium tremens | 🞏 Active alcohol abuse with social, behavioural or medical complications | 🞏 H/o alcohol abuse but not presently drinking |
| Illicit Drugs | 🞏 Acute Withdrawal Syndrome | 🞏 Active substance abuse with social, behavioural, or medical complications | 🞏 H/o substance abuse but not presently using |
| **Body Weight** | | | |
| Obesity |  | 🞏 Morbid (i.e., BMI ≥ 38) |  |

| **OVERALL COMORBIDITY SCORE (Circle one.)** | 0  None | 1  Mild | 2  Moderate | 3  Severe | 9  Unknown |
| --- | --- | --- | --- | --- | --- |

**Appendix 3: VES-13**

**VES-13**

1. Age: ______________________________
2. In general, compared to other people your age, would you say that your health is:

- Poor * (I POINT)

SCORE: 1 POINT FOR FAIR or POOR

- Fair* (I POINT)
- Good
- Very good, or
- Excellent

1. How much difficulty, on average, do you have with the following physical activities.

|  | No Difficulty | A little Difficulty | Some Difficulty | A Lot of Difficulty | Unable to do |
| --- | --- | --- | --- | --- | --- |
| 1. stooping, crouching, or kneeling? | 🞏 | 🞏 | 🞏 | 🞏* | 🞏* |
| 1. lifting or carrying objects as heavy as 10 pounds? | 🞏 | 🞏 | 🞏 | 🞏* | 🞏* |
| 1. reaching or extending arms above shoulder level | 🞏 | 🞏 | 🞏 | 🞏* | 🞏* |
| 1. writing or handling and grasping small objects? | 🞏 | 🞏 | 🞏 | 🞏* | 🞏* |
| 1. walking a quarter of a mile? | 🞏 | 🞏 | 🞏 | 🞏* | 🞏* |
| 1. heavy housework such as scrubbing floors or washing windows? | 🞏 | 🞏 | 🞏 | 🞏* | 🞏* |
|  | SCORE: 1 POINT FOR EACH * RESPONSE IN Q3a THROUGH Q3f. MAXIMUM OF 2 POINTS | | | | |

1. Because of your health or a physical condition, do you have any difficulty:
   1. Shopping for personal items (like toiletries or medicines)?

| 🞏 YES 🡪 Do you get help with shopping? | 🞏 YES* | 🞏 NO |
| --- | --- | --- |
| 🞏 NO |  |  |
| 🞏 DON’T DO 🡪 Is that because of your health? | 🞏 YES* | 🞏 NO |

- 1. Managing money (like keeping track of expense or paying bills)?

| 🞏 YES 🡪 Do you get help with managing money? | 🞏 YES* | 🞏 NO |
| --- | --- | --- |
| 🞏 NO |  |  |
| 🞏 DON’T DO 🡪 Is that because of your health? | 🞏 YES* | 🞏 NO |

- 1. Walking across the room? USE OF CANE OR WALKER IS OK.

| 🞏 YES 🡪 Do you get help with walking? | 🞏 YES* | 🞏 NO |
| --- | --- | --- |
| 🞏 NO |  |  |
| 🞏 DON’T DO 🡪 Is that because of your health? | 🞏 YES* | 🞏 NO |

- 1. Doing light housework (like washing dishes, straightening up, or light cleaning)?

| 🞏 YES 🡪 Do you get help with light housework? | 🞏 YES* | 🞏 NO |
| --- | --- | --- |
| 🞏 NO |  |  |
| 🞏 DON’T DO 🡪 Is that because of your health? | 🞏 YES* | 🞏 NO |

- 1. Bathing or showering?

| 🞏 YES 🡪 Do you get help with bathing or showering? | | | 🞏 YES* | 🞏 NO | |
| --- | --- | --- | --- | --- | --- |
| 🞏 NO | | |  |  | |
| 🞏 DON’T DO 🡪 Is that because of your health? | | | 🞏 YES* | 🞏 NO | |
| SCORE: 1 POINT FOR EACH * RESPONSE IN  Q4a THROUGH Q4e. |  | | |  |  |

**Appendix 4: Geriatric-8**

**Geriatric-8**

| **Items** | **Possible Answers** | **Score** |
| --- | --- | --- |
| Food intake in the last 3 months | 0: Severe reduction in food intake  1: Moderate reduction in food intake  2: Normal food intake |  |
| Weight loss during the last 3 months | 0: Weight loss >3kg  1: Does not know  2: Weight loss between 1 and 3 kg  3: No weight loss |  |
| Mobility | 0: Bed/Chair bound  1: Able to get out of bed/chair but does not go out  2: Goes out |  |
| Neuropsychological problems | 0: Severe dementia or depression  1: Mild dementia or depression  2: No psychological problems |  |
| Body Mass Index (BMI) | 0: BMI <19  1: BMI 19 to <21  2: BMI 21 to <23  3L BMI 23 or greater |  |
| Takes more than 3 medications per day | 0: Yes  1: No |  |
| Self-rated health status (compared to other people of the same age) | 0: Not as good  0.5: Does not know  1: As good  2: Better |  |
| Age | 0: >85  1: 80-85  2: <80 |  |
| Total Score (0-17)  [Cut-off ≤ 14 indicating impairment] |  | |
